# *Encephalitozoon intestinalis* Inhibits Dendritic Cell Differentiation through an IL-6-Dependent Mechanism

**DOI:** 10.3389/fcimb.2016.00004

**Published:** 2016-02-02

**Authors:** Carmen E. Bernal, Maria M. Zorro, Jelver Sierra, Katherine Gilchrist, Jorge H. Botero, Andres Baena, Jose R. Ramirez-Pineda

**Affiliations:** ^1^Grupo Inmunomodulación, Universidad de AntioquiaMedellín, Colombia; ^2^Grupo de Parasitología, Universidad de AntioquiaMedellín, Colombia; ^3^Grupo de Inmunología Celular e Inmunogenética, Universidad de AntioquiaMedellín, Colombia; ^4^Departamento de Microbiología y Parasitología, Universidad de AntioquiaMedellín, Colombia; ^5^Corporación Académica para el Estudio de Patologías Tropicales, Facultad de Medicina, Universidad de AntioquiaMedellín, Colombia

**Keywords:** *Encephalitozoon intestinalis*, microsporidia, dendritic cells, IL-6, IL-12

## Abstract

Microsporidia are a group of intracellular pathogens causing self-limited and severe diseases in immunocompetent and immunocompromised individuals, respectively. A cellular type 1 adaptive response, mediated by IL-12, IFNγ, CD4+, and CD8+ T cells has been shown to be essential for host resistance, and dendritic cells (DC) play a key role at eliciting anti-microsporidial immunity. We investigated the *in vitro* response of DC and DC precursors/progenitors to infection with *Encephalitozoon intestinalis* (*Ei*), a common agent of human microsporidosis. *Ei*-exposed DC cultures up-regulated the surface expression of MHC class II and the costimulatory molecules CD86 and CD40, only when high loads of spores were used. A vigorous secretion of IL-6 but not of IL-1β or IL-12p70 was also observed in these cultures. *Ei*-exposed DC cultures consisted of immature infected and mature bystander DC, as assessed by MHC class II and costimulatory molecules expression, suggesting that intracellular *Ei* spores deliver inhibitory signals in DC. Moreover, *Ei* selectively inhibited the secretion of IL-12p70 in LPS-stimulated DC. Whereas *Ei*-exposed DC promoted allogeneic naïve T cell proliferation and IL-2 and IFNγ secretion in DC-CD4+ T cell co-cultures, separated co-cultures with bystander or infected DCs showed stimulation or inhibition of IFNγ secretion, respectively. When DC precursors/progenitors were exposed to *Ei* spores, a significant inhibition of DC differentiation was observed without shifting the development toward cells phenotypically or functionally compatible with myeloid-derived suppressor cells. Neutralization experiments demonstrated that this inhibitory effect is IL-6-dependent. Altogether this investigation reveals a novel potential mechanism of immune escape of microsporidian parasites through the modulation of DC differentiation and maturation.

## Introduction

The phylum Microsporidia comprises a numerous group of obligate intracellular fungal parasites that infect a wide range of invertebrate and vertebrate animals. Formerly considered of only agricultural relevance, these organisms are increasingly recognized as important opportunistic pathogens causing severe diarrhea and systemic disease in HIV-infected individuals or organ-transplanted patients undergoing chemotherapy (Franzen and Müller, 2001; Didier and Weiss, 2006; Galván et al., 2011). Moreover, microsporidia are also common colonizers of immune-competent individuals in which self-limited or asymptomatic infections are observed (Ghosh and Weiss, 2012). In spite of being considered an emerging infectious disease for more than a decade, our knowledge about the mechanisms of pathogenesis and immunity during microsporidiosis is limited. Current understanding of the immune mechanisms characterizing and mediating the effective response to microsporidial infection has derived mostly from studies using the experimental infection of immune-deficient and immunocompetent mice with *Encehalitozoon cuniculi* (*Ec*). This model has indicated that (1) It is the cellular rather than humoral the important type of adaptive response that mediates protective immunity to Microsporidia; (2) IFNγ and IL-12, two cytokines known to mediate the development of the type 1 cellular response, are essential for protection; (3) an immune pathway mediated by CD8+ T cells via perforin-dependent cytotoxicity and IFNγ production appears to be the primary, and most important mechanism of protection, with the intestinal intraepithelial lymphocyte (IEL) CD8+ T cell compartment playing a key effector role; (4) in the absence of CD8+ T cells, a protective cellular response can be generated that is presumably mediated by IFNγ-producing CD4+ T cells (Ghosh and Weiss, 2012; Moretto et al., 2012). Some mice studies with the more common human pathogen *Encephalitozoon intestinalis* (*Ei*) have also revealed the importance of both CD4+ and CD8+ T cell populations as well as IFNγ and IL-12 production for host resistance (Salát et al., 2002, 2004).

Dendritic cells (DCs) are known as the most effective antigen presenting cells (APC), which prime naive T cells in order to become effector and memory cells (Steinman and Hemmi, 2006). Priming of Th1 and CD8+ T cells that mediate protection against intracellular infections is largely dependent on the IL-12 produced by DC in response to microbial signals (Trinchieri, 2003). Using an *in vitro* T cell priming system, Moretto et al. showed that only DC that were proficient to produce IL-12 in response to *Ec* were able to stimulate and expand Ag-specific naïve CD8+ T cells to become IFNγ producers and this result was consistent with the incapacity of IL-12-defficient mice to generate CD8+ T cells that express IFNγ and cytotoxic activity *in vivo* and that protect mice from lethal infection (Moretto et al., 2010). The ability of DC to prime CD8 T cells *in vitro* was dependent on the capacity of *Ec* to promote DC maturation and IL-12 production via TLR2 and TLR4 stimulation (Lawlor et al., 2010; Gigley and Khan, 2011). More strikingly, intestinal DC infected with *Ec* primed naïve IEL cells to proliferate *in vitro* and imprinted gut homing properties on spleen CD8+ T cells in an IFNγ-dependent manner (Moretto et al., 2007), demonstrating the importance of DC in the mucosal anti-microsporidian adaptive response. Recent developments in DC biology, however, indicate that microbial pathogens might interact in peripheral tissues not only with differentiated DC but also with DC precursors and progenitors in the steady-state and under inflammatory conditions and that the outcome of this interaction influences anti-microbial immunity (Massberg et al., 2007; Hespel and Moser, 2012). To gain a better understanding on the initial host's anti-microsporidian immune response, we exposed murine DCs and myeloid precursors to *Ei* spores *in vitro*, and analyzed the DCs differentiation and maturation process. We found that *Ei* spores are weak inducers of maturation on resting DC, and selective inhibitors of IL-12 secretion on maturing DC. In *Ei*-exposed DC cultures, bystander DCs were mature and promoted Ag presentation to naïve T cells whereas infected DCs were immature and inhibited T cell response. Exposure to *Ei* during DC differentiation inhibited the transformation of myeloid precursors into DC and this inhibition was dependent on the IL-6 present in the cultures. These results evidence novel immune escape mechanisms of microsporidia operating in this important leucocyte type.

## Materials and methods

### Animals

Six to nine weeks old female wild type BALB/c and C57BL/6 mice were obtained from Charles Rivers (Wilmington, MA). Mice were maintained in specific pathogen-free conditions. All animals were managed following the guidelines of the institutional ethical committee for animal experimentation (“Comité de ética para la experimentación con animales, Universidad de Antioquia, Medellín, Colombia”).

### *Ei* and DCs culture

*Ei* spores were kindly provided by Dr. A. Mathis (Institute of Parasitology, University of Zurich, Switzerland) and maintained by continuous passage in VERO cells, as previously reported (Didier et al., 1996). For some experiments, spores were labeled with carboxyfluorescein succinimidyl ester (CFSE; Invitrogen, Carlsbad, CA) as follows: 3 × 10^8^ spores were re-suspended in 1 ml PBS after two washing steps, incubated with 1 uL 0.5 mM CFSE, vortexed and incubated at 37°C for 10 min in the dark. The spores were then washed three times with PBS and re-suspended in complete culture medium RPMI 1640 (Glutamax™ Invitrogen, Carlsbad, CA) containing 10% FBS, 100 μg/ml streptomycin, 100 U/ml penicillin, and 50 μM 2β-mercaptoethanol. Labeling was confirmed by fluorescent microscopy and flow cytometry (Supplementary Figure 1A) and spores were immediately used for infections. DCs were generated from BALB/c bone marrow (BM) precursors as previously described (Lutz et al., 1999). mGM-CSF (Peprotech, New Jersey, USA) was added to BM cultures on days 0, 3, and 6 and cells in the supernatant were carefully collected on day 9 by decantation. Morphological, phenotypic and functional characteristics of the cells obtained with this protocol were confirmed as typical of DC (Supplementary Figure 2). Most reagents and solutions used for DC generation and culture were endotoxin-free as certified by manufacturers. In addition, samples from the different DCs and parasite cultures were periodically taken and tested for presence of endotoxin (which was < 0.1 EU/ml) by limulus assay (Limulus Amebocyte Lysate QCL-1000, Cambrex, Walkersville, MD).

### DCs infection

DCs were cultured at 1 × 10^6^/ml in the presence of *Ei* spores at different DC:parasite ratios and incubated for 24 h at 37°C with 5% CO_2_ atmosphere. Supernatants were then collected and frozen for subsequent measurement of cytokine release (IL-1β, IL-6, and IL-12p70) by ELISA. Cells were washed and either cytospun methanol-fixed and Giemsa-stained, or used to measure the surface expression of DCs maturation markers (CD40, CD86, and MHC class II) by flow cytometry. Giemsa-stained slides were analyzed by optical microscopy and the percentages of infected cells, as well as the average number of spores per 100 cells were registered. Non-treated or LPS (3 μg/ml, Sigma) -treated DCs were used as negative and positive maturation controls, respectively. In some experiments, DCs were infected during 6 h and subsequently treated with LPS for additional 18 h. In other experiments, the effect of infection on CD11c expression in DCs was determined. For the experiments where DCs were infected with CFSE-labeled spores, the discrimination of infected (CFSE-negative) or bystander (CFSE-negative) cells was confirmed by fluorescence microscopy and flow cytometry (Supplementary Figure 1B).

### Exposure to *Ei* spores during DCs differentiation

To determine the effect of *Ei* on DCs differentiation, a previously reported protocol (Nalbandian et al., 2005) was used with minor modifications. Briefly, 2 × 10^6^ BM cells were re-suspended in 10 ml culture medium containing rmGM-CSF and 3 days later 10 ml of fresh medium were added. For day 4, all non-adherent cells were harvested, re-suspended at 0.25 × 10^6^/ml and 2 ml seeded in 6-well plates. 1.25 ml fresh medium were then added and the cultures infected with *Ei* spores at different ratios. 1.25 ml medium per well were replaced by 1.25 ml fresh medium on the day 7 and, on day 9, cells in the supernatant were collected, counted and the number of CD11c+ cells determined by flow cytometry. BM cultures that were infected on day 4 and collected 24 h later were set in order to determine the cell viability by propidium iodide staining and flow cytometry, or to measure the levels of IL-1β, IL-6, and IL-10 in supernatants. In some experiments, day 9 cell cultures were treated with LPS (3 μg/ml), incubated for additional 24 h and the expression of CD11c and CD40, IL-1β, and IL-6 determined by flow cytometry and ELISA, respectively. In other experiments, DCs differentiation cultures were supplemented with rmIL-6 (eBioscience) and the effect on the production of CD11c+ cells determined. Likewise, some blocking experiments were performed with an anti-IL-6 mAb (eBioscience) in infected DCs differentiation cultures. Finally, to determine whether exposure of BM cells to *Ei* spores induces myeloid-derived suppressor cells (MDSC), cells were infected as above and the resulting cultures on day 9 were stained with anti CD11b, CD11c, and Gr1 Abs or used in T cell suppression assays. Non-treated or dexamethasone (Dexa, 3 μg/ml) -treated BM cultures were used as negative and positive controls, respectively.

### CD4+ T cell stimulation and suppression assays

Total spleen cells from naive C57BL/6 mice were obtained, red blood cell lysed with lysis buffer (Sigma, St. Louis, MO) and the CD4+ T cells purified by using a magnetic procedure (negative CD4+ T cell Isolation kit; Miltenyi, Cologne, Germany). Purity of preparations was always above 95%. *Ei*-infected DC were washed twice and the purified allogeneic CD4+ T cells were added at a 1:5 DC:T cell ratio. Co-cultures were incubated for 96 h and the concentration of IFNγ and IL-2 in the supernatant was determined by ELISA. In some co-cultures, cells were labeled with radioactive thymidine (^3^HTdR, 1 uCi/well) during the last 18 h of culture, and after harvesting, cell proliferation was assessed as cpm in a Plate Chameleon Microplate reader (Hydex, Turku, Finland). Some DC-CD4+ T cell co-cultures were performed with either *Ei*-infected DC or bystander DC. For this, DCs that were infected with CFSE-labeled *Ei* (MOI 30:1) for 24 h then were separated by fluorescent sorting (MOFLO XDP, Beckman Coulter, USA) as CFSE-positive or CFSE-negative DC and co-incubated with purified allogeneic CD4+ T cells during 72 h to finally quantify the amount of IFNγ released to the supernatant as an indicator of DC's Th1-inducing capacity. To determine whether myeloid cells differentiated in the presence of *Ei* had suppressive activity, BM cells were exposed to spores as described and titrated amounts of the resulting non adherent or adherent cell populations were incubated with CFSE-labeled (5 μM) BALB/c splenocytes polyclonally-activated with an anti-CD3e Ab (5 μg/ml, immobilized anti mouse CD3e, functional grade purified mAb, clone 145-2C11, eBioscience, San Diego, CA) plus IL-2 (10 ng/ml). After 72 h of culture, cells were collected, stained with an anti-CD4 mAb and analyzed by flow cytometry. T cell proliferation was reported as the percentage of cells with diluted CFSE among CD4+ cells.

### Flow cytometry and ELISA

Cells were blocked at 4°C for 10 min with FBS and for 10 min with purified rat anti-mouse CD16/CD32 (clone 2.4G2). The cells were then stained with the following antibodies: PE-labeled anti CD40 (clone 3/23), PE-labeled anti CD86 (GL-1), biotin-conjugated anti IA^d^ (clone AMS-32.1), PE-Cy7-conjugated anti CD11c (clone HL-3), PE-conjugated anti-CD11b (clone M1/70), and FITC-conjugated Ly6G and Ly6C (Gr1) (clone CRB6-8C5). PE-Cy5-conjugated streptavidin was used as second antibody where it applies. A PE-Cy5-conjugated anti mouse CD4 (clone GK1,5) was also used for T cells staining. All these antibodies, as well as the isotype-matched controls, were from BD Biosciences (Franklin Lakes, NJ). Samples were acquired in an Epics XL or a Beckman Coulter Cytomix FC500 flow cytometer (Beckman Coulter, Brea, CA). Analysis was performed using the WinMDI (version 2.8) or Summit (version 3.1) software. The levels of IL-1β, IL-2, IL-6, IL-10, IFNγ, and IL-12p70 were determined by using ELISA kits (BD OptEIA ELISA kits, BD Biosciences, Franklin Lakes, NJ, USA) following manufacturer recommendations. The detection limits were 31.3, 3.1, 15.6, 31.3, 3.1, and 62.5 pg/ml for IL-1β, IL-2, IL-6, IL-10, IFNγ, and IL-12p70, respectively.

### Statistical analysis

Three to four replicate wells were performed for each culture condition where statistical analysis was intended. The mean +∕− SEM were calculated and graphed. The surface maturation marker levels were reported as the MFI from 10,000 events. ANOVA analysis with Newman Keuls test, Bonferroni's test, or student's *t*-test was applied to evaluate the statistical significance of differences among groups. Graphs and analysis were performed in the GraphPad PRISM 5.0 software.

## Results

### The DC response to infection with *Ei* spores

**Figure 1:**
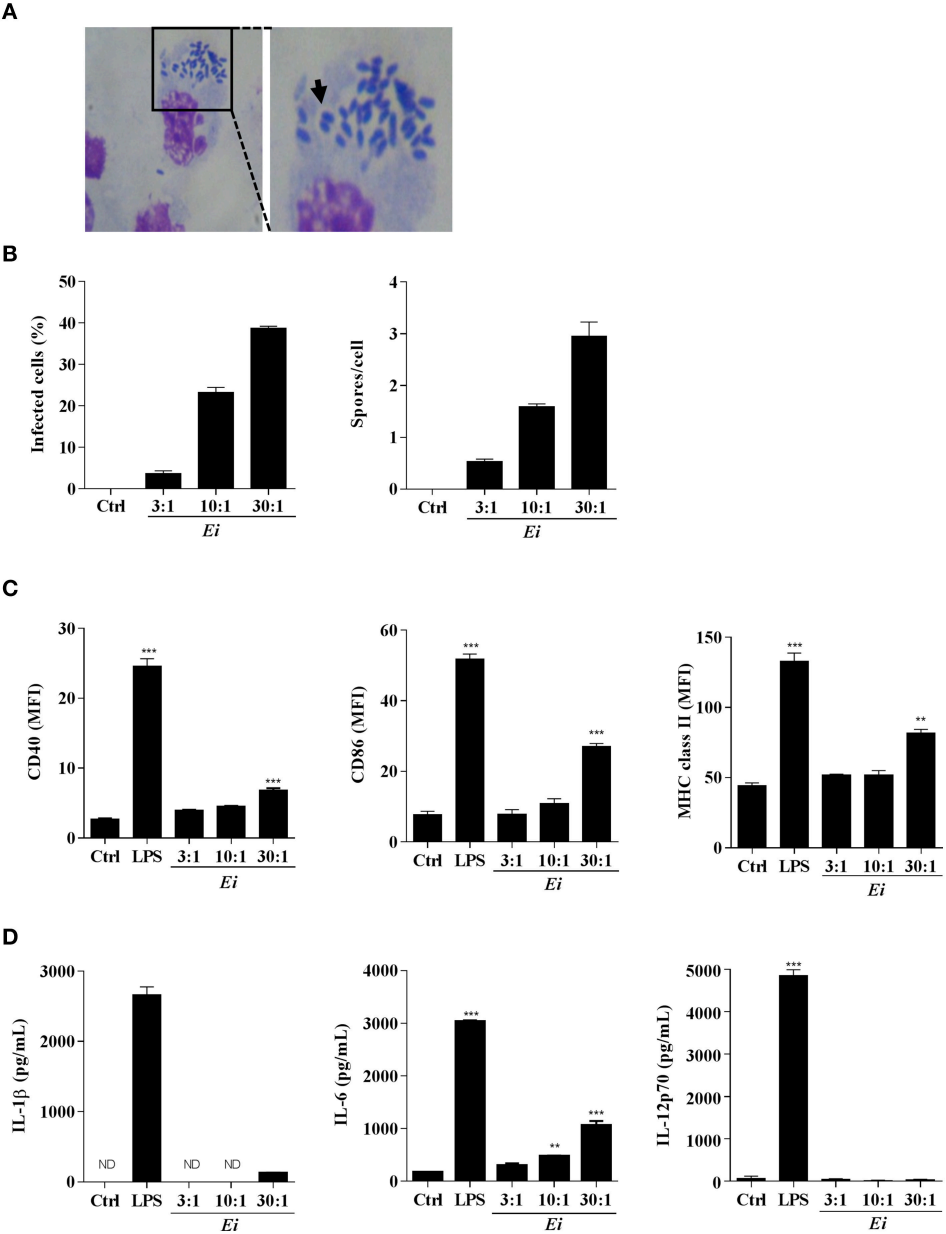
**Resting DCs respond to infection with *Ei* by expressing surface maturation markers and secreting IL-6**. DCs were generated and cultured in presence of *Ei* spores at the indicated MOI for 24 h. Cells were fixed on glass slides, Giemsa-stained and observed under optical microscope. A representative microphotograph of infected cultures (30:1 MOI) is shown **(A)**. The arrow shows a parasitophorous vacuole containing *Ei* spores. The percentage of infected cells as well as the number of spores per DC was evaluated and graphed **(B)**. DCs were also evaluated for the surface expression of CD40, CD86, and MHC class II molecules by flow cytometry **(C)**. Abundance of surface markers is expressed as the MFI. The concentrations of IL-1β, IL-6, and IL-12p70 secreted to the supernatants were also evaluated by ELISA **(D)**. Non-treated or LPS-treated cells were used as negative or positive maturation controls, respectively. Data are shown as the mean ± SEM (triplicate cultures). ANOVA test: ^**^p < 0.01; ^***^p < 0.001, compared to the control group. ND, not detected. Magnifications in A: 20X and 40X.

We first wanted to determine whether DCs could internalize *Ei* spores under our culture conditions. After infection with *Ei* spores for 24 h, the typical vacuoles containing spores were observed in Giemsa-stained DC preparations (Figure 1A). When we measured the percentage of infected cells and the number of spores per DC, we found that both proportionally increased with the multiplicity of infection (MOI; Figure 1B). Moreover, spores were viable at 24, 48, and 72 h post-infection, as demonstrated by their ability to infect and lyse VERO cell monolayers (not shown). Then, we determined the capability of *Ei* spores to induce DCs maturation by measuring the surface expression of CD40, CD86 and MHC class II and the secretion of the proinflammatory cytokines IL-1β, IL-6, and IL-12p70 (Figures 1C,D). Results shown in Figure 1C indicate that *Ei* is a weak inducer of DC maturation, as only very high loads of *Ei* spores were able to significantly up-regulate the different maturation markers as compared to the non-infected control (see also Supplementary Figure 3 for representative histograms). Even at the highest MOI, the surface abundance of maturation markers in *Ei*-exposed DC never reached the levels observed in LPS-treated cells (Figure 1C; Supplementary Figure 3). Likewise, *Ei* promoted the secretion of IL-6 by DC in a dose-dependent and significant manner, but not of IL-1β or IL-12p70 (Figure 1D). DCs were healthy and responsive, as they expressed high levels of all maturation markers and cytokines after stimulation with LPS (Figures 1C,D). Thus, these results show that *Ei* spores are able to infect DC in a relatively silent manner, at low and moderate MOIs, and that only the exposure to high dose of infection (30:1 parasite to cell ratio) could promote a relatively weak but significant level of maturation characterized by increased expression of CD40, CD86, MHC class II and IL-6 but non-detected/low levels of IL-1β and IL-12p70.

**Figure 2:**
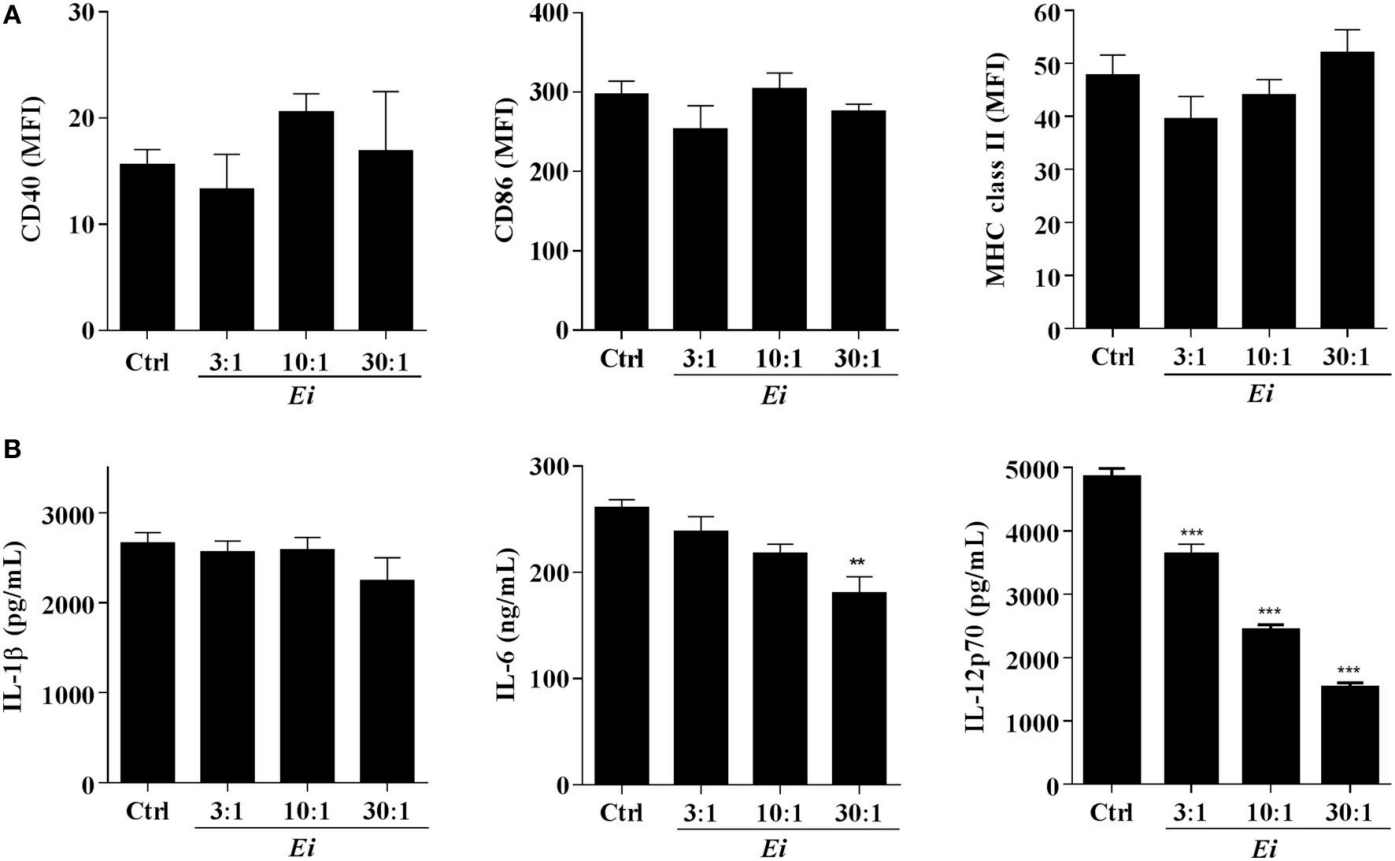
***Ei* inhibits LPS-induced IL-12p70 production by DC**. DCs were cultured in presence of *Ei* spores during 6 h at the indicated MOI and subsequently treated with LPS for additional 18 h. Cell pellets were used to quantify the surface expression of CD40, CD86, and MHC class II molecules by flow cytometry **(A)** and the supernatants were used to measure the concentration of IL-1β, IL-6, and IL-12p70 by ELISA **(B)**. The surface markers abundance is expressed as the MFI. DCs treated with LPS only were used as controls. Data are shown as the mean ± SEM (triplicate cultures). ANOVA test: ^**^p < 0.01; ^***^p < 0.001, compared to the control group.

The high MOIs required to induce some DC maturation by *Ei* spores prompted us to investigate a possible inhibitory effect. Thus, DCs were infected with *Ei* at different MOIs and subsequently activated with LPS (Figure 2). When surface maturation markers were measured no differences in the expression of CD40, CD86, or MHC class II were found between infected and non-infected DC (Figure 2A). Interestingly, the measurements of cytokines showed that whereas the *Ei*-infected DCs were competent to secrete IL-1β and IL-6, they were dose-dependently inhibited to secrete IL-12p70 (Figure 2B). Although *Ei* appears to slightly limit DC's ability to secrete IL-1β and IL-6 at high MOIs, cells still produced high levels of these cytokines. In contrast, IL-12 was significantly inhibited even at low MOI. Altogether, these findings reveal intriguing effects of *Ei* on these cells: low level of maturation and IL-6 production in resting DC, but selective interference with the production of the Th1-promoting cytokine IL-12 in maturing DC.

### T cell activation by *Ei*-exposed DC

**Figure 3:**
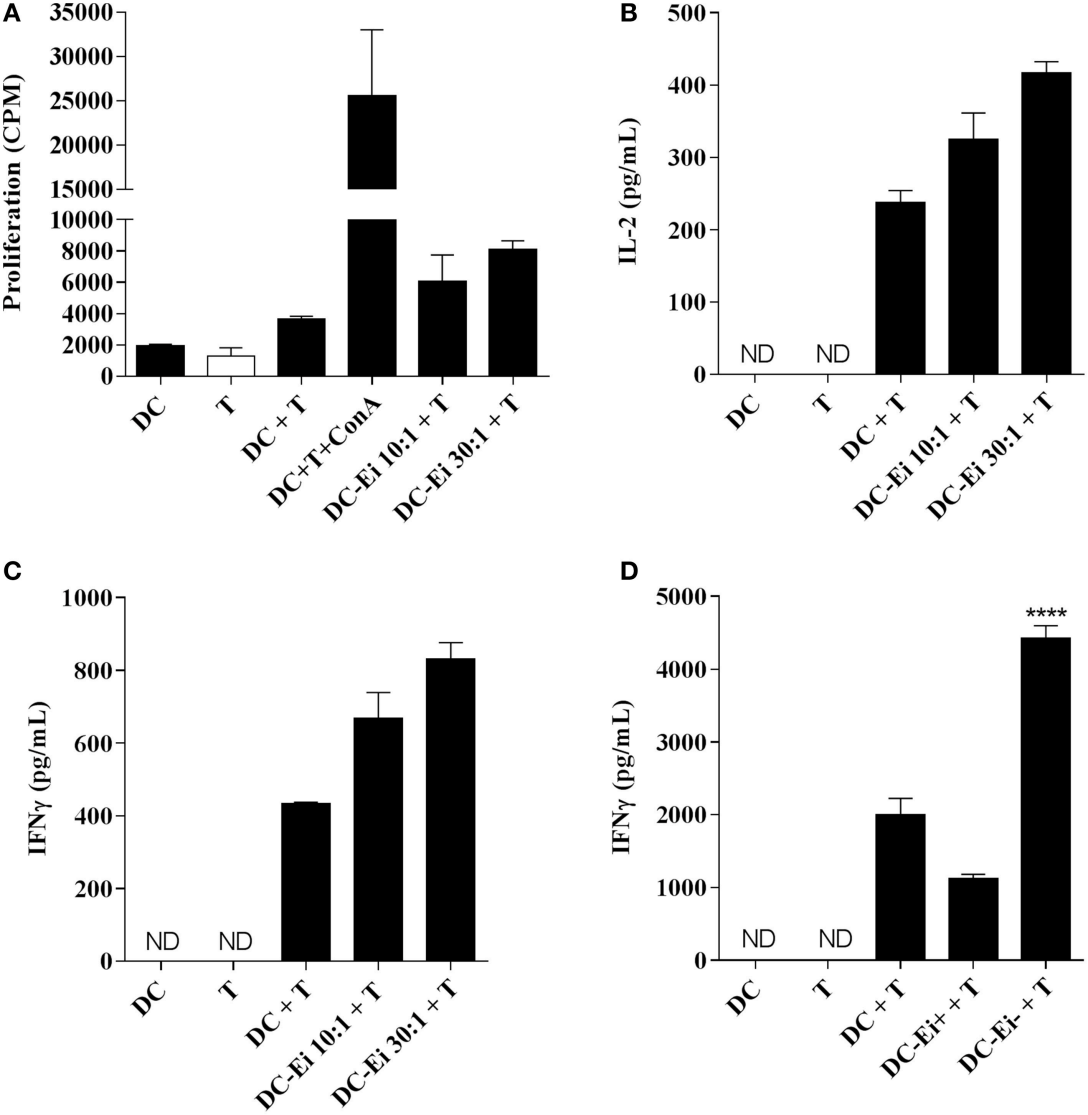
**APC function of DC is modulated by *Ei* infection**. DC were cultured in the presence of *Ei* spores at the indicated MOI during 24 h and subsequently co-incubated with purified allogeneic naïve CD4+ T cells for additional 96 h. The proliferation **(A)** as wells as the amount of IL-2 **(B)** and IFNγ **(C)** secreted to the supernatants was determined by ^3^H-TdR incorporation and ELISA, respectively. DC-CD4+ T cell co-cultures were also set with sorted CFSE-positive infected or CFSE-negative bystander DC and the production of IFNγ after 72 h incubation was determined **(D)**. Data are shown as the mean ± SEM (duplicate cultures in **A–C**, triplicate cultures in **D**). ANOVA test: ^****^p < 0.0001, compared to the control non-infected co-culture group. ND, not detected.

In order to explore the functional consequences of the *Ei*-DCs interaction on T cells, allogeneic naive CD4 T cells were co-incubated with DC cultures that were previously exposed to *Ei* spores and the proliferation as well as the secretion of IL-2 and IFNγ was measured. Figure 3 indicates that DC cultures exposed to *Ei* were competent APC, as they were able to promote both the proliferation (Figure 3A), IL-2 (Figure 3B), and IFNγ production (Figure 3C) of naive CD4+ T cell in a dose-dependent manner. The stimulation of proliferation and IL-2 production by CD4+ T cells was consistent with our previous observations showing that *Ei*-infected DC cultures not only up-regulated surface molecules important for Ag presentation (Figure 1C), but also were responsive to further stimulus (Figure 2). However, the production of IFNγ was unexpected, based on the evident inhibitory effect of *Ei* infection on LPS-induced IL-12p70 secretion (Figure 2B).

As we showed before, we notice that even at the highest MOI tested, the DCs cultures contained a high proportion of non-infected bystander cells (Figure 1B). For this reason, it was important to determine which population of DCs (infected or bystander), changed the levels of expression of maturation markers. For this purpose, DCs cultures were exposed to CFSE-labeled spores and infected cells were discriminated from bystanders as CFSE-positive and CFSE–negative, respectively (Supplementary Figure 1B). As shown in the Supplementary Figure 4, cells expressing high level of the maturation markers are mostly present in the bystander population, whereas infected DCs express lower amounts of these markers. These data suggest that DC cultures exposed to *Ei* spores consist of a major population of maturing bystander cells and a second population of immature DCs harboring parasites. Moreover, in accordance with results shown in Figure 2A, both infected and bystander DCs were not refractory to further stimulation, as they up-regulated surface maturation markers after LPS treatment in a comparable manner to non-infected DC (not shown). Interestingly, whereas bystander DC promoted IFNγ production, infected DC were incapable to induce similar levels of IFNγ production by allo-reactive naïve CD4 T cells (Figure 3D), indicating important functional differences between those DC populations in their Th1-activating capacity.

In summary, the results of our experiments in differentiated DC indicate that (1) whereas IL-6 production and low level of maturation characterize the DC response to *Ei* under resting conditions, selective inhibition of IL-12 production characterizes the response to infection under activating conditions; (2) Exposition of DC to *Ei* spores, results in both infected cells and non-infected “bystanders,” that exhibit segregated levels of maturation; (3) whereas mature bystanders DC promote, immature infected DC inhibit IFNγ secretion by naïve T cells.

### *Ei* interferes with DCs differentiation from myeloid precursors

**Figure 4:**
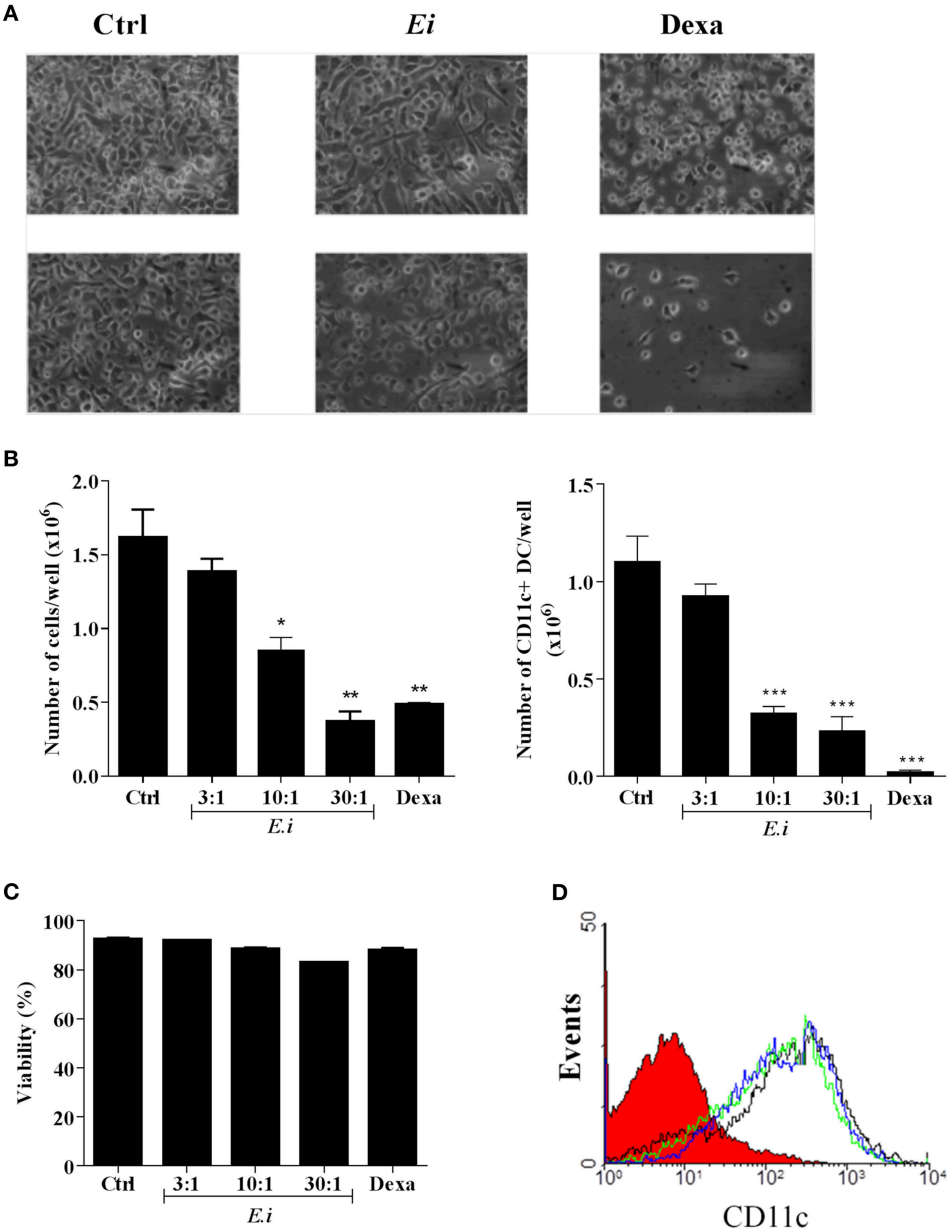
***Ei* interferes with the differentiation of DCs from myeloid precursors**. BM cells were cultured with GM-CSF for 4 days and *Ei* spores were added (at different MOI, as indicated). Non-treated or Dexamethasone-treated cultures were set as negative and positive controls, respectively. Cells were kept in GM-CSF-supplemented culture medium to complete 9 days. Representative images of cultures (*Ei*:cells 30:1; Dexa 3 μg/ml) photographed with inverted microscope are shown before (upper panel) and after (lower panel) removing non-adherent cells **(A)**. Cells in supernatants were collected, counted, and stained with an anti CD11c mAb to determine de percentage of DC (CD11c+ cells) by flow cytometry. Results are presented as the total number of cells (**B**, left) or the number of CD11c+ DCs (**B**, right) per well. The viability of BM cells after 24 h treatments was also determined by propidium iodide staining and flow cytometry **(C)**. The effect of *Ei* on the CD11c expression was also determined in differentiated DC (9-days GM-CSF-supplemented BM cultures) after 24 h infection. Representative histograms of DC infected with a MOI of 10:1 (blue line), 30:1 (green line), or non-treated DC (black line) are shown **(D)**. The histogram of an isotype-matched Ab is shown in red. Results are shown as the mean ± SEM (*n* = 3−5 for **B**, *n* = 2 for **C**). ANOVA test: ^*^p < 0.05; ^**^p < 0.01; ^***^p < 0.001, compared to the control.

**Figure 5:**
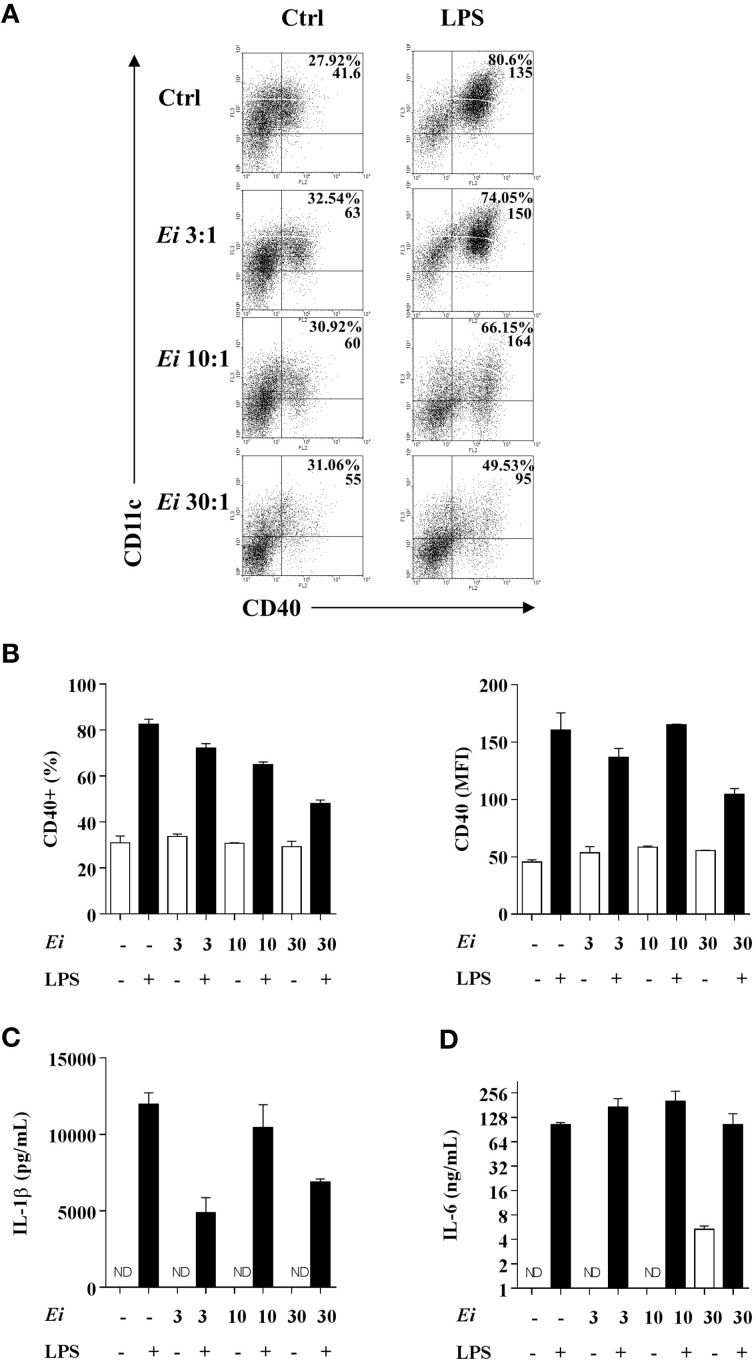
**DCs differentiated in the presence of *Ei* respond to LPS stimulation**. GM-CSF-supplemented BM cultures were set and infected with *Ei* spores at different MOI, as indicated. After 9 days of differentiation cells were transfer to new wells and treated with LPS during 24 h. Cells were used to quantify the surface expression of CD40 by flow cytometry. Representative dot-plots of CD40 vs. CD11c markers are shown **(A)**. Numbers in dot-plots indicate the percentage and MFI of the indicated quadrant. The percentages of CD40+ cells gated from the CD11c+ population (**B**, left) as well as the MFI for the CD40 marker in the CD11c+ CD40+ population (**B**, right) were graphed. The supernatants of the same cultures were used to measure the concentration of the cytokines IL-1β and IL-6 by ELISA **(C,D)**. Data are shown as the mean ± SEM from duplicate cultures. ND, not detectable.

We also investigated whether the infection with *Ei* influences DCs differentiation. For this, GM-CSF-supplemented BM cultures were taken at day 4, infected with *Ei* spores and re-incubated for 5 more days to finally evaluate the amount of CD11c+ cells present at day 9. Our first observation, as shown in representative images of the cultures (Figure 4A), was a reduction in the amount of cells in the wells exposed to *Ei* compared to control cultures. Dexamethasone (Dexa), used as a positive control (Rozkova et al., 2006), dramatically reduced the adherent and non-adherent cell yield in BM cultures (Figure 4A). When cells in the supernatants were counted and analyzed by flow cytometry, we noticed that *Ei* infection significantly reduced the total amount of cells and the absolute numbers of CD11c+ cells in a dose-dependent manner (Figure 4B), without affecting the non-adherent cell population (Supplementary Figure 5A). Exposure to *Ei* did not affect cell viability in BM cells (Figure 4C), or CD11c expression in differentiated DC (Figure 4D), suggesting that the reduction in CD11c+ cells was a consequence of differentiation inhibition rather than toxicity or CD11c down-regulation. Interestingly, when DC differentiated in the presence of *Ei* were treated with LPS, the surface expression of the maturation marker CD40 was significantly up-regulated (Figures 5A,B). Although, the number of CD11c+ cells that developed at the highest MOI was reduced as compared to the control (Figure 4B), they were still able to mature in response to the treatment with LPS (Figures 5A,B). Furthermore, quantification of IL1-β and IL-6 in the supernatants confirmed that *Ei*-conditioned DCs were responsive to LPS maturation (Figures 5C,D). Altogether, our results demonstrate that *Ei* inhibits DC differentiation from myeloid precursors without affecting their responsiveness to further maturation stimuli.

**Figure 6:**
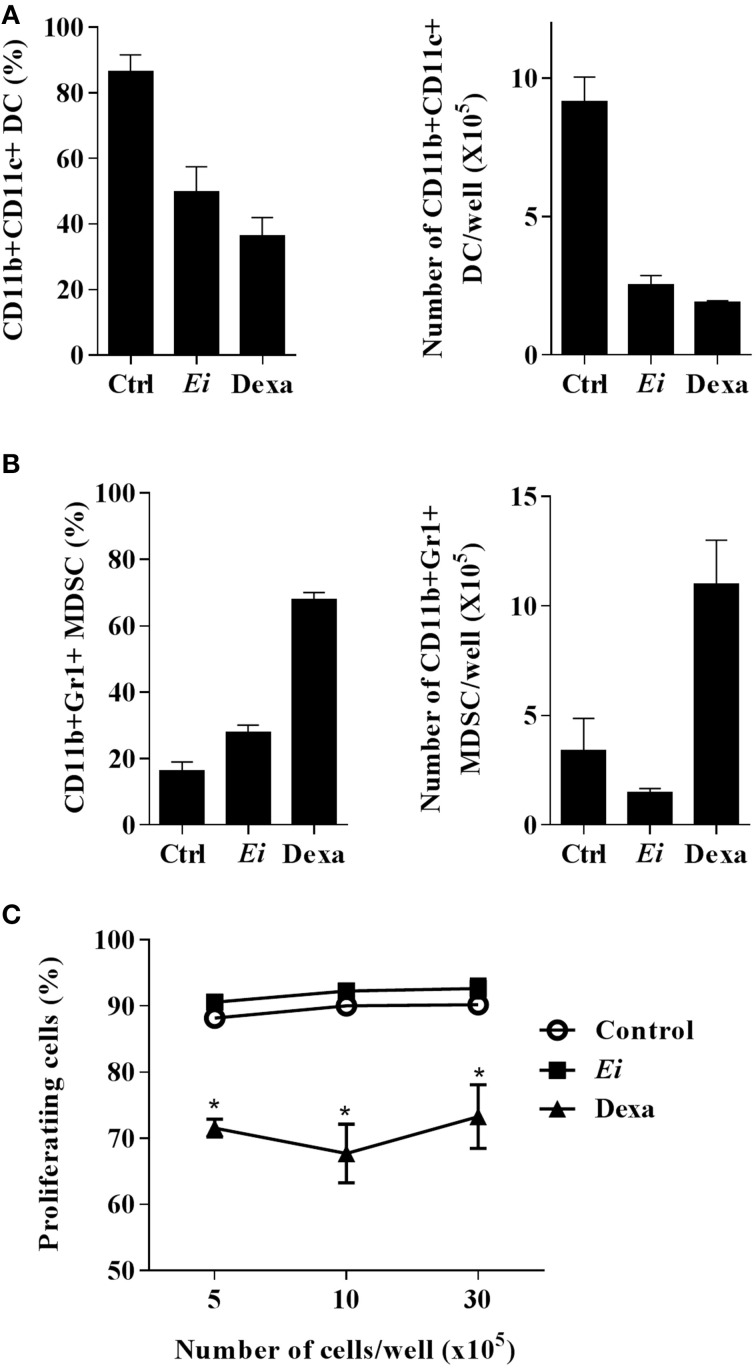
***Ei* inhibit DC differentiation but does not promote MDSC formation**. BM cells were cultured with GM-CSF for 4 days and *Ei* spores were added (MOI of 30:1). Non-treated or Dexa-treated cultures (day 4) were set as negative and positive controls, respectively. Cells were kept in GM-CSF-supplemented culture medium to complete 9 days. Cells in supernatants were then collected, counted and stained with an anti CD11b, CD11c, and Gr1 mAbs to determine de amounts of CD11b+ CD11c+ Gr1- DCs **(A)** or CD11b+ CD11c- Gr1+ MDSC **(B)** by flow cytometry. Results are presented as the percentage (left) or the absolute number per well (right). The indicated amounts of cells per well were also co-cultured with polyclonally-activated (anti CD3 mAb/rIL-2) CFSE-labeled naïve lymph node cells during 72 h. The percentage of cells with diluted CFSE was then determined by flow cytometry to assess the suppressive effect **(C)**. Graphs show the mean ± SEM (*n* = 2–3 for **A** and **B**, *n* = 4 for **C**). Student's *t*-test: ^*^p < 0.05, compared to control.

GM-CSF and IL-6 are recognized as potent inducers of MDSC differentiation (Marigo et al., 2010). Our finding that *Ei* promotes IL-6 secretion in myeloid precursors (Figure 5D) and in DCs (Figure 1D) prompted us to explore the possibility that this parasite shifted the differentiation program of BM precursors from DC to MDSC. BM cells were cultured and infected with *Ei*, as above, and the production of CD11b+ CD11c+ Gr1- cells (DC) or CD11b+ CD11c- Gr1+ cells (MDSC) was monitored. Non-treated or Dexa-treated cultures were used as negative and positive controls, respectively. Results in Figures 6A,B show that whereas Dexa inhibited the differentiation of DC and promoted the generation of cells phenotypically compatible with MDSC, *Ei* exhibited only the former effect. Since MDSC are more operationally than phenotypically defined (Haile et al., 2012), it was important to confirm whether *Ei*-conditioned BM cultures exhibited suppressive activity. Dexa- or *Ei*-exposed BM cultures were collected on day 9 and the resulting non-adherent cells were used to evaluate their suppressive capacity in polyclonally-activated T cells. As expected, Dexa-conditioned cells suppressed the proliferation of CD4+ T cells (Figure 6C). In contrast, CD4+ T cell proliferation was not affected by BM cultures conditioned with *Ei* (Figure 6C). Moreover, adherent cells that were always present in *Ei*-conditioned BM cultures (Figure 4A) were also phenotyped and used in similar suppression assays, and no induction of MDSC was observed (Supplementary Figure 5). Thus, results indicate that *Ei* spores inhibit DC differentiation without promoting MDSC formation.

### The inhibitory effect of *Ei* on DC differentiation is IL-6-dependent

**Figure 7:**
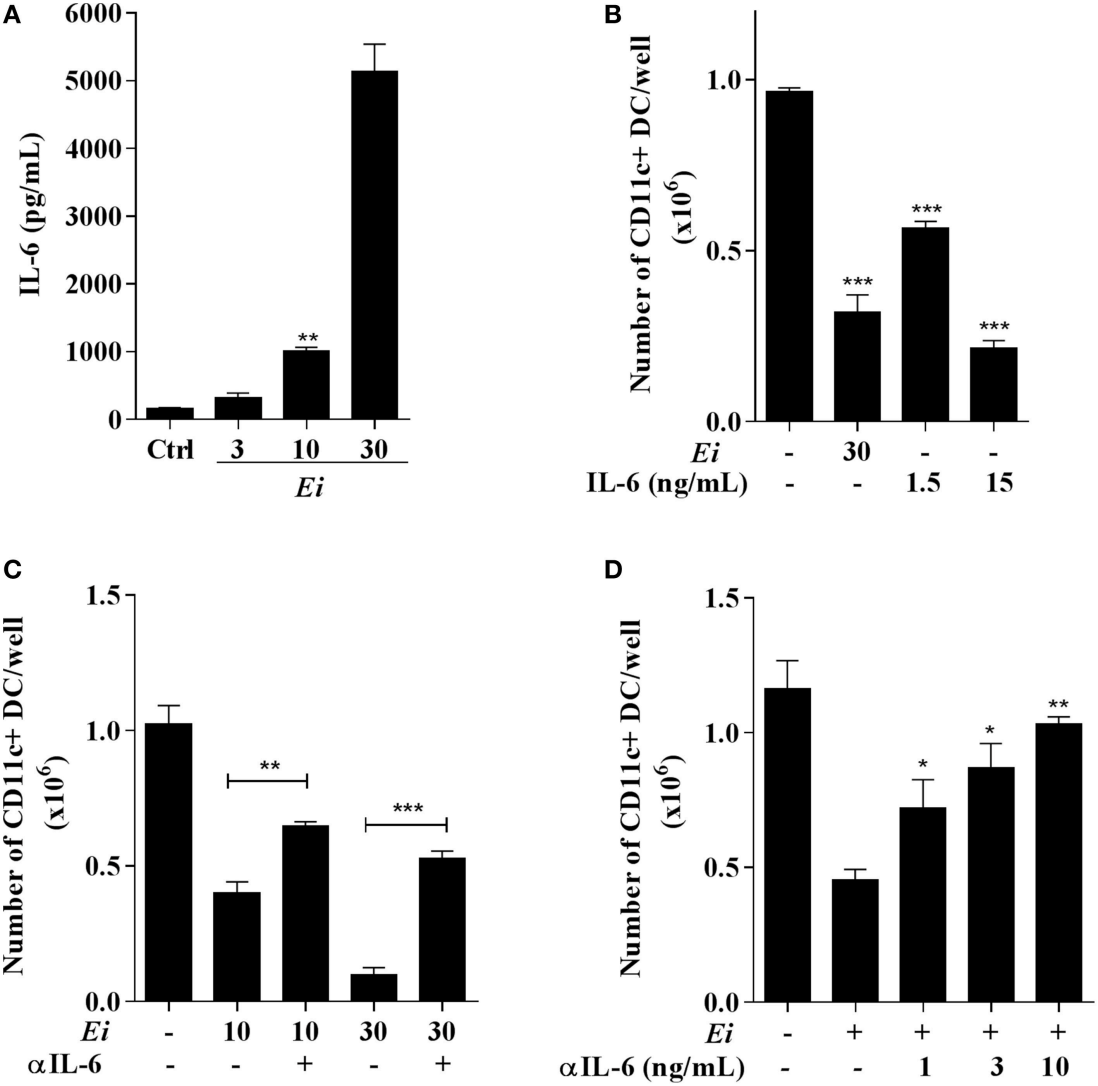
**Inhibition of DC differentiation by *Ei* spores is IL-6-dependent**. BMDC cultures were set and at day 4 cells were exposed to *Ei* spores (at the indicated MOI). Twenty four hours later the supernatants were collected and the concentration of IL-6 determined by ELISA **(A)**. Similarly, the effect of rIL-6 (1.5 o 15 ng/ml) addition to cultures (also at day 4) was compared to *Ei* infection **(B)**. Blocking experiments with fixed (5 ng/ml; **C**) or graded **(D)** amounts of an anti-IL-6 mAb added at days 4 and 5 were also performed. In B, C and D cells were collected at day 9 and assessed for surface CD11c expression in order to calculate the total number of CD11c+ cells per well. Data are the mean ± SEM (triplicates). ANOVA test: ^*^p < 0.05, ^**^p < 0.01, ^***^p < 0.001, compared to the control in **(A)**, to non-treated/non-infected in **(B)**, to the indicated group in **(C)** and to non-treated/infected in **(D)**.

Based on the previous results where BM-derived DC differentiation cultures infected with a high dose of *Ei* spores and in the absence of LPS stimulation accumulated significant amounts of IL-6 in the supernatants (Figure 5D), we proposed a role for this cytokine in the observed inhibitory effect. To establish this, we took BM precursors and exposed them overnight to different MOI of *Ei* spores in order to quantify the secretion of IL-6, IL-1β and IL-10. Consistently with our previous results, a dose-dependent secretion of IL-6 (Figure 7A) and not detectable levels of IL-1β (data not shown) were observed in this experiment after 24 h incubation. Moreover, no detectable secretion of IL-10, an anti-inflammatory cytokine triggered by many microbial pathogens and implicated in DC differentiation inhibition (Allavena et al., 1998), was observed in the same supernatants (not showed). High concentrations of IL-6 were observed in the supernatants during the entire culture period (data not shown), indicating that DC precursors were permanently exposed to this cytokine. To further explore the potential role of IL-6 as an inhibitory cytokine for DC differentiation, BM cultures were supplemented with rIL-6 in addition to GM-CFS and compared to *Ei*-infected cultures. We found that the exposure of BM cells to IL-6 inhibited the differentiation of CD11c+ cells in a dose-dependent manner (Figure 7B). BM cultures exposed to rIL-6, with concentrations that resemble those found in *Ei*-exposed BM cultures, mimicked the inhibitory effect of Ei spores (Figure 7B). These results indicate that IL-6 is sufficient to prevent DC differentiation in a comparable manner to *Ei* infection, which further implicates this cytokine as a key player in this effect. To confirm this hypothesis, BM cells were infected with 10:1 and 30:1 MOI in the presence of neutralizing anti-IL-6 antibodies. As showed in Figure 7C the blocking of IL-6 significantly recovered the numbers of CD11c+ cells in the cultures. Furthermore, the inhibitory effect of *Ei* could be completely abrogated by increasing the amounts of the neutralizing anti-IL-6 Ab (Figure 7D). Thus, we demonstrated that IL-6 is responsible for the inhibition of DC differentiation induced by *Ei*.

## Discussion

Recent developments in microsporidian genomics have emphasized the exceptional adaptation of these organisms to intracellular parasitism (Vávra and Lukeš, 2013). The presence of latent infection in immune-compromised, and more strikingly, in immune-competent individuals (Ghosh and Weiss, 2012), implies that microsporidian parasites have evolved mechanisms to avoid or modulate the host's immune responses and thus assure survival and persistence. Previous studies have shown that DCs are important for immune recognition and activation against microsporidia (Moretto et al., 2007, 2010; Lawlor et al., 2010). Here we report for the first time that *Ei*, one of the most important causal agents of human microsporidiosis, modulates both DC differentiation and maturation *in vitro*, and this might represent an important immune evasion mechanism exhibited by these parasites.

We found that *Ei* infected DC in a silent manner when low MOI were used. However, when spore loads were high, cells responded by up-regulating surface maturation markers and secreting IL-6, but not other inflammatory cytokines like IL-1β or IL-12. Our results are consistent with the report of Lawlor et al. (2010) in which a relatively weak induction of MHC class II and costimulatory molecules was observed in splenic DC infected with Ec, and additionally extend them by showing that microsporidia can stimulate IL-6 secretion on resting DC. Whereas it is not surprising that a threshold in parasite loads has to be reached before DCs are able to sense infection, it is intriguing that *Ei* selectively induced IL-6 secretion. We performed a further cytokine analysis by using a sensitive multiplex Luminex technology and confirmed that IL-6 was by far the most abundant cytokine secreted in *Ei*-infected DC cultures (data not shown). In Lawlor's study the production of IL-6 or IL-1β was not evaluated but an increased frequency of IL-12p40-producing splenic DCs was reported after *Ec* infection *in vivo* and *in vitro*. Although the production of the secreted bioactive IL-12p70 was not demonstrated in that study, the physiological consequences of its absence were probed (Lawlor et al., 2010). Although in other study using a per-oral model of *Ec* infection IFNγ production by intestinal DC was not documented, IFNγ-expressing DCs were required to prime T cells *ex vivo* (Moretto et al., 2007). In the absence of complete information on the different cytokines expressed in those experimental settings, a simple explanation for all these results is that the profile of cytokines produced by resting DCs in response to microsporidian infection is dependent on the parasite species and the type of DC involved (e.g., lymphoid resident, tissular migratory or inflammatory). This is not surprising, considering the diversity of microsporidian parasites (Pombert et al., 2013) and the phenotypical and functional complexity of the dendritic cells system (Merad et al., 2013).

Of similar interest was the finding that *Ei* selectively interferes with the secretion of IL-12p70 in DC activated by a subsequent stimulus. Notably, whereas surface markers were not affected and a slight inhibitory trend was seen in IL-1β and IL-6 secretion at the highest MOI (Figure 2), a pronounced reduction (~70%) in IL-12p70 secretion was consistently observed. Even at the lowest MOI used, IL-12p70 secretion was significantly impaired, suggesting an adaptation of *Ei* to specifically inhibit this Th1-inducing cytokine. Although a functional inactivation that makes DC refractory to further maturation stimuli has been reported after infection with viral (Gredmark-Russ and Söderberg-Nauclér, 2012), bacterial (Hanekom et al., 2003), protozoal (McKee et al., 2004), and fungal parasites (Huston et al., 2013; Zimmer et al., 2011), the selectivity observed in our study is unprecedented to the best of our knowledge. In spite that IL-12 production is primarily triggered by microbial ligands via PRR, other signals such as those delivered by activated T cells or cytokines are also important to boost IL-12 response in DC (Spörri and Reis e Sousa, 2003). Whether *Ei* interferes with the IL-12 secretion promoted by non-microbial signals (such as CD40L) was not investigated here. However, our antigen presentation assays (Figure 3D) suggested that this might be the case. Since studies in other infectious models have associated alterations in JAK/STAT, NF-kB, IFNγ regulatory factor (IRF), and phosphoinositide 3-kinase (PI3K) signaling pathways to DC inhibition (Xin et al., 2008; Kamda and Singer, 2009) future efforts should confirm if these or other cellular and molecular mechanisms are linked to the *Ei*-mediated DC dysfunction reported here. A recent report that used a quantitative proteomic approach identified variations in the abundancy of proteins related to redox balance/signaling and IFNs response in microsporidia-infected human fibroblasts (Panek et al., 2014), suggesting the identity of particular pathways potentially implicated in *Ei*-triggered DC maturation dysfunction. Likewise, as shown for other microbial pathogens (Geijtenbeek et al., 2003; Kawai and Akira, 2011), it will be important to identify the activating microsporidian ligands and DC receptors triggering stimulation of resting DC, as well as those mediating inhibition in maturing DCs. Although some evidence has implicated TLR2, TLR4, and the MyD88 adapter in innate recognition of microsporidia (Fischer et al., 2008; Zhang et al., 2011), much needs to be explored to clarify the contribution of the different pathogen recognition receptors (PRR). The vast amount of information available on the innate receptors and pathways utilized by fungal pathogens in mammalian hosts will guide rapid progress in this field.

In an infected tissue not all the cells exposed to the intracellular infectious agent are actually infected. These non-infected bystanders, although devoid of the direct effects emanating from the hosted microbe, are physiologically influenced by factors released by the pathogen or the infected cells. Recent reports using bacterial infection in epithelial cells have suggested that bystander innate activation may have a greater practical importance than previously appreciated (Hardt, 2010). We could investigate how DC maturation was induced in bystander vs. infected cells, since even at MOIs as high as 30:1 only 40% of *Ei*-exposed cells had internalized spores (Figure 1B). Because intracellular cytokine staining offered a low signal-to-noise ratio and did not provide information on the bioactive form of the mediators, we focused our analysis on DC maturation as indicated by the surface expression of MHC class II and costimulatory molecules. In agreement with our results in bulk DC cultures (Figure 1C), we found that both infected and bystander DC responded by up-regulating maturation markers. However, bystanders were more mature than infected cells (Supplementary Figure 4). A similar pattern of mature bystanders and less mature infected DC has been observed in DC exposed to other intracellular pathogens such as *Leishmania* (Carvalho et al., 2008) and dengue virus (Palmer et al., 2005). Given that infected cells and bystanders are exposed to the same extracellular influences, results indicated that intracellular *Ei* spores deliver inhibitory maturation signals to DC. These inhibitory signals, however, operate only in resting DC, as they were clearly overcome when further activation was applied to bulk DC cultures (Figure 2A) or sorted bystander and infected DC (not shown). This contrasted with the sustained inhibition observed in *Leishmania*- and dengue virus-infected DCs where no increase in surface maturation markers was demonstrated after LPS, or TNFα stimulation, respectively (Palmer et al., 2005; Carvalho et al., 2008). Thus, whereas maturation appears permanently altered in DCs invaded with those primary pathogenic viral and protozoal organisms, it can be rescued through further maturation stimulus in DC infected with the opportunistic microsporidia *Ei*. It remains to be determined whether this is a consistent difference between primary and opportunistic pathogens at the interphase DC-microbe. However, what can be concluded from our results is that Microsporidia establishes a more balanced and less suppressive host-parasite relationship with DC.

As could be concluded from the antigen presentation assays to CD4+ T cells, the modulatory effects of *Ei* on DC maturation and cytokine production had important repercussions on the adaptive response. Bulk *Ei*-exposed DCs were able to stimulate allogeneic naïve CD4+ T cell proliferation and cytokine production, including IFNγ (Figures 3A–C). This was in agreement with the studies using Ec-exposed bulk DC from the spleen and the intestine co-incubated with syngeneic CD8+ T cells (Moretto et al., 2007, 2010; Lawlor et al., 2010). However, when bystanders and infected DC were sorted and separately co-cultured with T cells the IFNγ concentration in the supernatants was stimulated or inhibited, respectively (Figure 3D). Thus, we believe that whereas bystander DC are good APC that prime CD4 T cells to proliferate and initiate an IFNγ response, parasitized DCs are inhibited to do so because their ability to up-regulate IL-12 production in response to T-cell-derived signals is impaired. Although additional experiments need to be performed to confirm this hypothesis, our results indicate that upon infection with *Ei* spores two qualitatively different DC subpopulations are formed, one of mature bystander cells that are capable to promote IFNγ production in naive CD4+ T cells and other of less mature infected DC with the opposite effect on T cells. This dichotomy in the functional properties of infected vs. bystander DC was formerly proposed by Scott and collaborators in a model of dermotropic leishmaniosis (Carvalho et al., 2008). In this case, bystander DC promoted T cell response whereas infected DCs were poor T cell activators but potent producers of TNFα, an immune-pathologically relevant pro-inflammatory cytokine. More recently, in a viscero-tropic *Leishmanial* model, mature IL-12p40- and IL-6-producing bystander DC induced protective T cell response whereas less mature IL-10-and TNFα-producing infected DC induced non-protective IL-10-producing effector Th1 cells, typically associated with parasite persistence (Resende et al., 2013). Since T cell response to microsporidia is also characterized by IL-10 production (Khan and Moretto, 1999), it will be interesting to evaluate if *Ei*-parasitized DC are able to promote IL-10-producing T cells (Mathews et al., 2009). Moreover, taking into account the important role of CTL response in microsporidian infections, it is tempting to propose that bystanders vs. infected DC might generate qualitatively different CD8+ T cell responses. In the context of the pro-inflammatory potential of *Ei*-exposed DC, a question remains of whether bystander or infected DCs are the IL-6 producers and how this affects T cell responses. Our preliminary experiments blocking IL-6 during DC-T cell co-cultures did not show a pronounced effect on T cell activation as measured by IFNγ secretion (not shown). Although a more detailed analysis of the immune consequences of microsporidian infections in DC should be performed in the future, this study is the first to reveal the effects of *Ei* on this important leucocyte type and provides new insights on the complexity of this parasitic disease.

Whereas the effects of microbial pathogens on DC maturation have been extensively investigated, the influence of microbial exposure on DC differentiation has been less documented. Myelopoiesis and DC production might be influenced directly by invasion of the BM by pathogens (Welner et al., 2008), or indirectly through soluble mediators released by pathogens or infected/injured host cells (Nagai et al., 2006). Moreover, during infection in the periphery, pathogens may encounter not only resident differentiated DC, but also tissue resident or infiltrating DC precursors, and even earlier hematopoietic stem cells and progenitors including DC progenitors (Massberg et al., 2007). Furthermore, of special interest has been the demonstration *in vivo* that during infection, infiltrating monocytes transform into DC *in situ*, and that these emerging inflammatory DCs play an important role in innate and adaptive responses (Hespel and Moser, 2012). Because GM-CSF-elicited DCs are recognized as *in vitro* counterparts of inflammatory DCs generated *in vivo* (Zhan et al., 2012), we took advantage to investigate whether *Ei* influences DC differentiation from myeloid precursors by exposing differentiating BMDC to the spores. Interestingly, we observed a dramatic reduction in the DC yield when BM cultures were infected with *Ei* (Figure 4). Thus, *Ei* appears to subvert an inflammatory DC developmental pathway leading to a reduced pool of potential Th1-inducing APC. The relevance of our finding is highlighted by the studies reporting that inflammatory DCs are important for antimicrobial immune response in several tissues including intestinal mucosa (Hespel and Moser, 2012). Moreover, because GM-CSF is also critical for the development of a resident intestinal DC subset in the steady-state (Bogunovic et al., 2009) that mediated resistance to microsporidian infection (Moretto et al., 2007), it is possible that *Ei* interferes with the development of both migratory intestinal DC in addition to inflammatory DC. Pathogens have been described as promoters of DC differentiation (Cao et al., 2012; Hou et al., 2012), and recent reports implicated several PRR expressed in DC precursors/progenitors as triggers of this process (Krutzik et al., 2005; Massberg et al., 2007; Schmid et al., 2011; Yáñez et al., 2011; Merad et al., 2013; Miles et al., 2013). Increasing evidence, however, has revealed specific adaptations of viral (Carlier et al., 2011; Guo et al., 2012; Inagaki et al., 2012), bacterial (Pastille et al., 2011; Remoli et al., 2011), fungal (Torosantucci et al., 2004; Nisini et al., 2007), and parasitic (Skorokhod et al., 2004; Kanan and Chain, 2006; Favali et al., 2007; Riganò et al., 2007; Wykes et al., 2007; Fujiwara et al., 2009; Mejri et al., 2011; Markikou-Ouni et al., 2012) organisms to subvert this process as a strategy of immune escape. The interfering effect exhibited by these microorganisms usually led to regulatory DC or to a disabled form of APC that does not adequately respond to maturation stimulus and/or does not efficiently stimulate T cells (Torosantucci et al., 2004; Kanan and Chain, 2006; Nisini et al., 2007; Riganò et al., 2007; Wykes et al., 2007; Fujiwara et al., 2009; Mejri et al., 2011; Pastille et al., 2011; Guo et al., 2012; Markikou-Ouni et al., 2012). This contrasts with the inhibition by *Ei* reported here, in which the resulting DC pool was quantitatively reduced, but cells reaching the CD11c+ status were readily responsive to LPS maturation (Figure 5). This again, evidences a more equilibrated strategy of immune evasion in microsporidia as compared to the more immunosuppressive strategy observed with those other cited pathogens.

The mechanisms leading to microbial-induced inhibition of DC differentiation are poorly understood. Reports showing that this effect can be reproduced with microbial components (Nisini et al., 2007; Riganò et al., 2007), suggest a direct role for PAMPs presumably via PRR on the DC precursors/progenitors. However, because some cytokines such as IL-1β, IL-6, or IL-10 typically produced during infection are known to alter DC differentiation (Allavena et al., 1998; Chomarat et al., 2000; Makino et al., 2006), indirect mechanisms might be of similar importance. Indeed, *Mycobacterium tuberculosis* was shown to stimulate p38 MAP kinase- and STAT-3-dependent signaling pathways in human monocytes leading to the increased IL-10 secretion, which in turn, inhibited the transformation of bystander monocytes into DC (Remoli et al., 2011). Also, human cytomegalovirus infection in monocytes leads to increased IL-6 secretion, the paracrine induction of SOCS3 and the subsequent inhibition of STAT5 phosphorylation, which is a key transducer of the GM-CSF receptor and therefore of monocyte to DC differentiation signals in both infected and bystander cells (Carlier et al., 2011). We found that DC precursors in *Ei*-infected BMDC differentiating cultures were chronically exposed to high levels of IL-6 but not to any measurable IL-1β or IL-10 and that IL-6 was both sufficient and necessary for the observed *Ei*-mediated inhibition of DC differentiation (Figure 7). This demonstrated that IL-6 but no other cytokine is responsible for the observed effect. Whether SOCS3 and altered GM-CSF signaling is involved in the inhibition of DC differentiation by *Ei* reported here is not known at this point but appears an interesting possibility that requires future investigation. A comparative proteomic analysis in infected vs. non-infected human fibroblasts revealed that altered abundancies in JAK-STAT and Ras signaling proteins make part of the microsporidia-host cell “cross-talk” (Panek et al., 2014). Although the levels of activated JAK and Ras remain to be determined, the key role of these signal transducers for myeoid cell proliferation and differentiation make them good candidates for future mechanistic explorations of the *Ei*-induced inhibition of DC differentiation reported here. Our results, however, demonstrate that a non-viral pathogen also exploits IL-6 signaling to subvert DC differentiation. We speculate that DC, macrophages and myeloid precursors and progenitors present in our BMDC cultures produce IL-6 upon *Ei* sensing, and this in turn inhibits DC differentiation in a paracrine manner. Moreover, in the *in vivo* situation, epithelial cells may be a robust source of IL-6 after microsporidian infection. Indeed, preliminary experiments have evidenced that an intestinal epithelial cell line produce profuse amounts of IL-6 after *Ei* infection (not shown). Thus, by inducing preferential expression of IL-6 in epithelial cells, myeloid precursors and differentiated DC and macrophages, *Ei* may delay the onset of the adaptive response allowing a temporal advantage that favors microbial multiplication and persistence.

Given the preponderant role of IL-6 during *Ei* infection together with the observation that GM-CSF and IL-6 are well-recognized promoters of MDSC (Marigo et al., 2010), we evaluated whether *Ei* shifted the differentiation of BM cells from DC to MDSC. However, we could not demonstrate any phenotypic or functional evidence for such a shift in our *Ei*-exposed cultures in both non-adherent (Figure 6) and adherent cells (Supplementary Figure 5). Thus, once more *Ei* appear to be a good evader of the relevant immune response without promoting immunosuppression, although this could be more evident in aged mice and could involve additional molecules such us TGF-β and PDL-1 (Mathews et al., 2009; Gigley and Khan, 2011; Bhadra et al., 2014).

In summary, we presented *in vitro* evidence indicating that *Ei* evades the immune system by interfering with several aspects of the DC physiology: (1) provoking a silent infection when the microbial load is low, and thus, the parasite avoids early recognition and guaranties replication. (2) Once the microbial loads grow and recognition by DC is imminent, *Ei* promotes a low level of DC maturation and the selective production of IL-6 but no or very little production of other pro-inflammatory cytokines. This assures a limited amplification of the innate response, and a modulated transmission of activating signals to the adaptive system. Moreover, IL-6 secretion by myeloid cells leads to inhibition of DC differentiation from precursors, limiting the available pool of APC able to initiate a protective anti-*Ei* adaptive response. (3) Interfering selectively with the signals leading to the production of the IL-12 in activating DC, further limiting IFNγ production by T cells. Since IFNγ-producing T cells are a key host resistance factor against microsporidia, *Ei* limits the relevant response required for its control. Although future work should confirm the relevance *in vivo* of these findings, our work provides new insights into the microsporidia-mammalian host relationship that improves our understanding of the immune-biology of this emerging infectious agent.

## Author contributions

CB and JR designed the study. CB, MZ, JS, and KG conducted experiments. JB and AB contributed with reagents or data analysis. JR and AB wrote the manuscript.

## Funding

This work was supported by Colciencias (Grants 1115-343-19225; 1115-519-28906; 1115-569-33796) and Universidad de Antioquia (CODI CPT-0411; CPT-0607; CPT-1217).

### Conflict of interest statement

The authors declare that the research was conducted in the absence of any commercial or financial relationships that could be construed as a potential conflict of interest.

## Supplementary material

Supplementary Figure 1 **Fluorescent labeling of *Ei* spores and discrimination of infected and bystander DC**. *Ei* spores were labeled with CFSE as described in methodology. Fluorescent staining of parasites was confirmed by fluorescence microscopy (**A**, left) and flow cytometry (**A**, right). Histograms with red or green lines represent non-labeled control and CFSE-labeled spores, respectively. CFSE-labeled spores were also used to infect DC (30:1 MOI) and the resulting culture was stained for surface CD86 (red) and nucleus (DAPI, blue). A representative fluorescent microscopy photograph of an *Ei*-infected cell is shown (40X magnifications; **B**, left). Flow cytometric analysis of these DC cultures also permitted to discriminate infected spores (I, CFSE-positive, purple dots) from non-infected (or bystanders, B, CFSE-negative, black dots) DC (**B**, right). I and B gates were based on dot-plots from DC infected with non-labeled spores.

Supplementary Figure 2 **The morphological, phenotypycal and functional characteristics of BMDC used in this study are typical of DC**. BMDCs were generated as described in the methodology section and non-adherent cells were taken for microscopic, cytometric and functional characterization. A typical image taken with inverted microscope at low magnification (10X) from a day-9 DC culture shows typical clusters of loosely adherent DC on a monolayer of firmly adherent macrophages (**A**, left). A closer view (40X) of a non-adherent cell in the same culture permitted to confirm the typical DC morphology as a rounded cell with long cytoplasmic processes or dendrites (**A**, middle). Giemsa-stained slides from supernatants also confirmed the production of a homogeneous population of cells with rounded or indented peripheral nuclei (**A**, right). The surface phenotype analyzed by FACs was also characteristic of DC **(B)**. DCs from BALB/c mice were, as expected, potent APC when co-cultured with allogeneic (C57BL/6) T cells **(C)**. Finally, a divergent ROS response of non-adherent DC and adherent macrophages to oxLDL stimulation confirmed the differential functional characteristics of those BM-derived cell populations. ROS production was assessed fluorometrically by using the probe 2,7-diclorofluorescein diacetate. VERO cells were used as a control of non-myeloid cells **(D)**. oxLDL, oxidized low density lipoprotein; Mo, macrophage; RFU, relative fluorescent units.

Supplementary Figure 3 **Representative histograms showing the surface expression of CD40, CD86 and MHC class II on DC cultures infected with *Ei***. DCs were generated and cultured in the presence of *Ei* spores at the indicated MOI for 24 h and the surface expression of CD40, CD86, and MHC class II molecules was quantified by flow cytometry. Non- or LPS-treated DCs were used as negative and positive controls, respectively. Filled histograms are from the isotype controls.

Supplementary Figure 4 **In *Ei*-exposed cultures bystander DCs are more mature than infected DCs**. DCs were cultured in presence of CFSE-labeled *Ei* spores at the indicated MOI for 24 h and the surface expression of CD40, CD86, and MHC class II molecules was quantified by flow cytometry in the CFSE-negative (bystander, B) and the CFSE-positive (infected, I) populations. Non-treated DCs were used as negative controls. The numbers on the histograms represent the MFIs for each marker.

Supplementary Figure 5 **Myeloid cell precursors exposed to *Ei* do not develop into adherent MDSC**. BM cells were cultured with GM-CSF for 4 days and *Ei* spores were added (MOI of 30:1). Non-treated or Dexa-treated cultures (day 4) were set as negative and positive controls, respectively. Cultures were kept in GM-CSF-supplemented culture medium to complete 9 days. Cells in supernatants were then removed and the adherent cells collected, counted and stained with an anti CD11b, CD11c, and Gr1 mAbs to determine de amounts of CD11b+ CD11c+ Gr1- DCs **(A)** or CD11b+ CD11c- Gr1+ MDSC **(B)** by flow cytometry. Results are presented as the percentage (left) or the absolute number per well (right). The indicated amounts of cells per well were also co-cultured with polyclonally-activated (anti CD3 mAb/rIL-2) CFSE-labeled naïve lymph node cells during 72 h. The percentage of cells with diluted CFSE was then determined by flow cytometry to assess the suppressive effect **(C)**. Graphs show the mean ±SEM (*n* = 2 for **A** and **B**, *n* = 4 for **C**). Student's *t*-test: ^*^*p* < 0.05, compared to control.

## Abbreviations

APC: Antigen presenting cells
BM: Bone marrow
CFSE: carboxyfluorescein diacetate succinimidyl ester
DC: Dendritic cells
Dexa: Dexamethasone
*Ec*: *Encephalitozoon cuniculi*
*Ei*: *Encephalitozoon intestinalis*
IEL: Intestinal intraepithelial lymphocyte
MDSC: Myeloid-derived suppressor cells
MOI: multiplicity of infection
^3^HTdR: Radioactive thymidine.
