# Supplementary figures and images for: Encephalitozoon intestinalis Inhibits Dendritic Cell Differentiation through an IL-6-Dependent Mechanism
Source: Front Cell Infect Microbiol. 2016 Feb 2;6:4. doi: 10.3389/fcimb.2016.00004 (PMC4735406; doi:10.3389/fcimb.2016.00004)

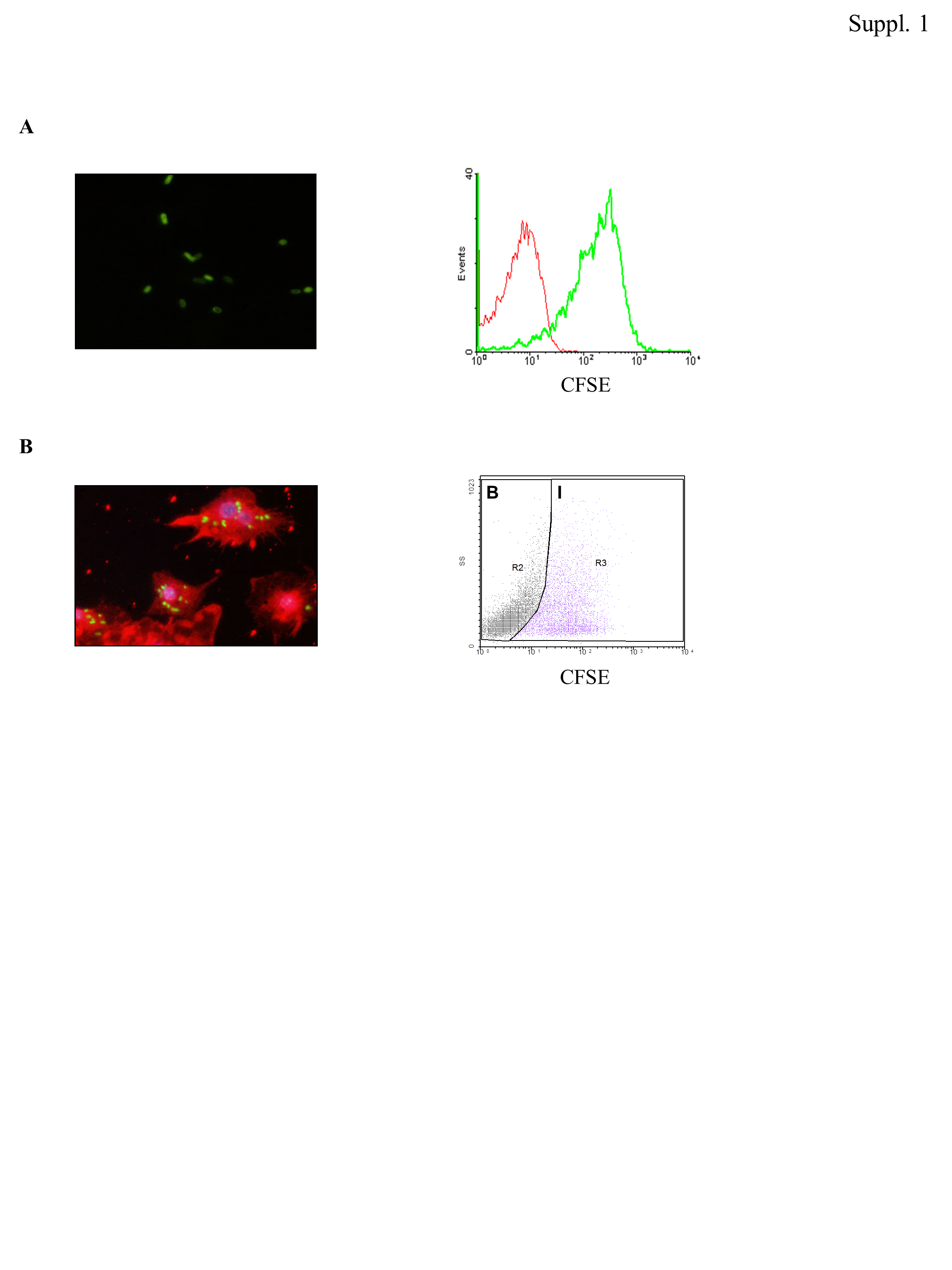

Supplement: Supplementary Figure 1 — Fluorescent labeling of Ei spores and discrimination of infected and bystander DC. Ei spores were labeled with CFSE as described in methodology. Fluorescent staining of parasites was confirmed by fluorescence microscopy (A, left) and flow cytometry (A, right). Histograms with red or green lines represent non-labeled control and CFSE-labeled spores, respectively. CFSE-labeled spores were also used to infect DC (30:1 MOI) and the resulting culture was stained for surface CD86 (red) and nucleus (DAPI, blue). A representative fluorescent microscopy photograph of an Ei-infected cell is shown (40X magnifications; B, left). Flow cytometric analysis of these DC cultures also permitted to discriminate infected spores (I, CFSE-positive, purple dots) from non-infected (or bystanders, B, CFSE-negative, black dots) DC (B, right). I and B gates were based on dot-plots from DC infected with non-labeled spores. [file Image1.TIF]

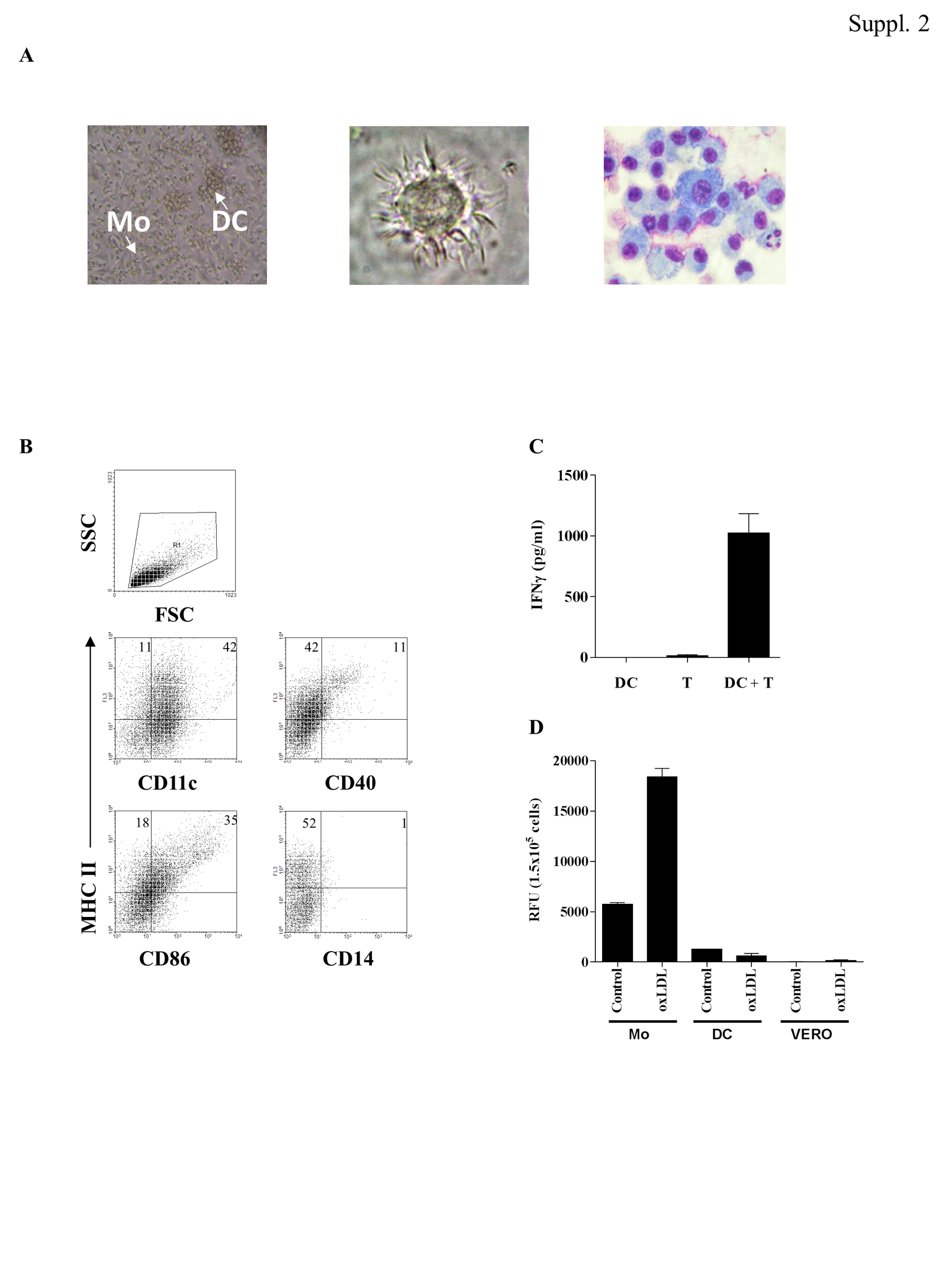

Supplement: Supplementary Figure 2 — The morphological, phenotypycal and functional characteristics of BMDC used in this study are typical of DC. BMDCs were generated as described in the methodology section and non-adherent cells were taken for microscopic, cytometric and functional characterization. A typical image taken with inverted microscope at low magnification (10X) from a day-9 DC culture shows typical clusters of loosely adherent DC on a monolayer of firmly adherent macrophages (A, left). A closer view (40X) of a non-adherent cell in the same culture permitted to confirm the typical DC morphology as a rounded cell with long cytoplasmic processes or dendrites (A, middle). Giemsa-stained slides from supernatants also confirmed the production of a homogeneous population of cells with rounded or indented peripheral nuclei (A, right). The surface phenotype analyzed by FACs was also characteristic of DC (B). DCs from BALB/c mice were, as expected, potent APC when co-cultured with allogeneic (C57BL/6) T cells (C). Finally, a divergent ROS response of non-adherent DC and adherent macrophages to oxLDL stimulation confirmed the differential functional characteristics of those BM-derived cell populations. ROS production was assessed fluorometrically by using the probe 2,7-diclorofluorescein diacetate. VERO cells were used as a control of non-myeloid cells (D). oxLDL, oxidized low density lipoprotein; Mo, macrophage; RFU, relative fluorescent units. [file Image2.TIF]

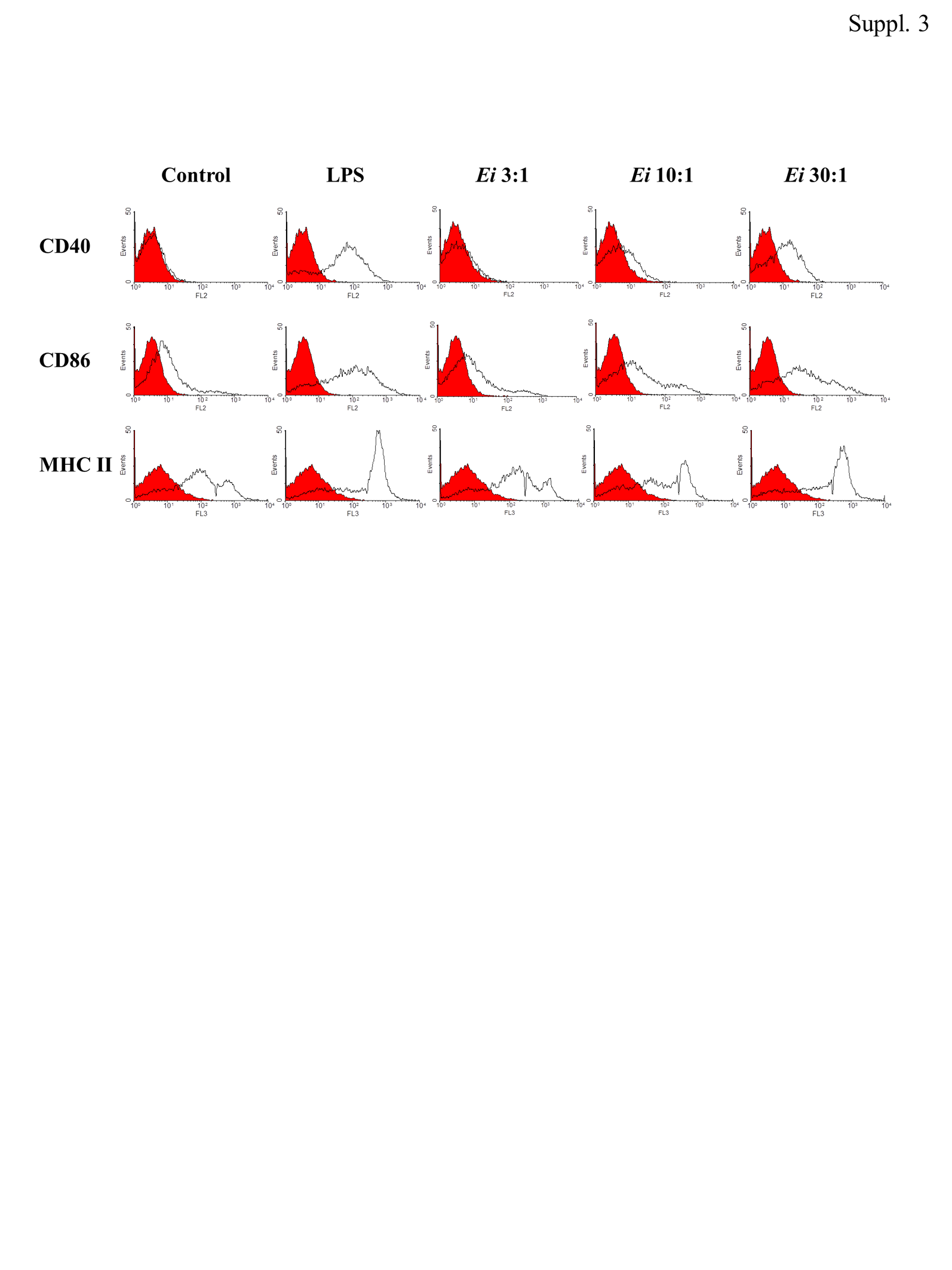

Supplement: Supplementary Figure 3 — Representative histograms showing the surface expression of CD40, CD86 and MHC class II on DC cultures infected with Ei. DCs were generated and cultured in the presence of Ei spores at the indicated MOI for 24 h and the surface expression of CD40, CD86, and MHC class II molecules was quantified by flow cytometry. Non- or LPS-treated DCs were used as negative and positive controls, respectively. Filled histograms are from the isotype controls. [file Image3.TIF]

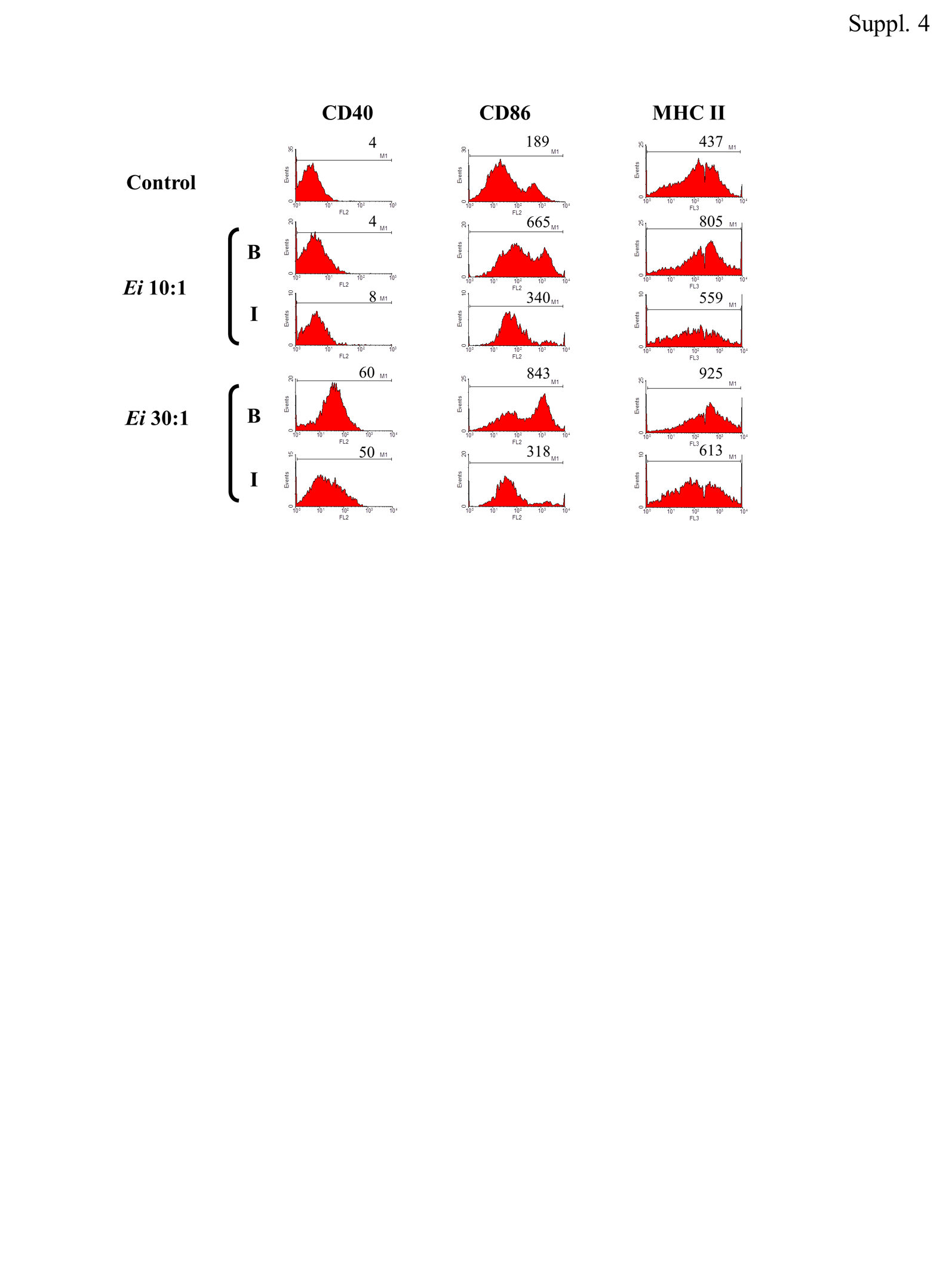

Supplement: Supplementary Figure 4 — In Ei-exposed cultures bystander DCs are more mature than infected DCs. DCs were cultured in presence of CFSE-labeled Ei spores at the indicated MOI for 24 h and the surface expression of CD40, CD86, and MHC class II molecules was quantified by flow cytometry in the CFSE-negative (bystander, B) and the CFSE-positive (infected, I) populations. Non-treated DCs were used as negative controls. The numbers on the histograms represent the MFIs for each marker. [file Image4.TIF]

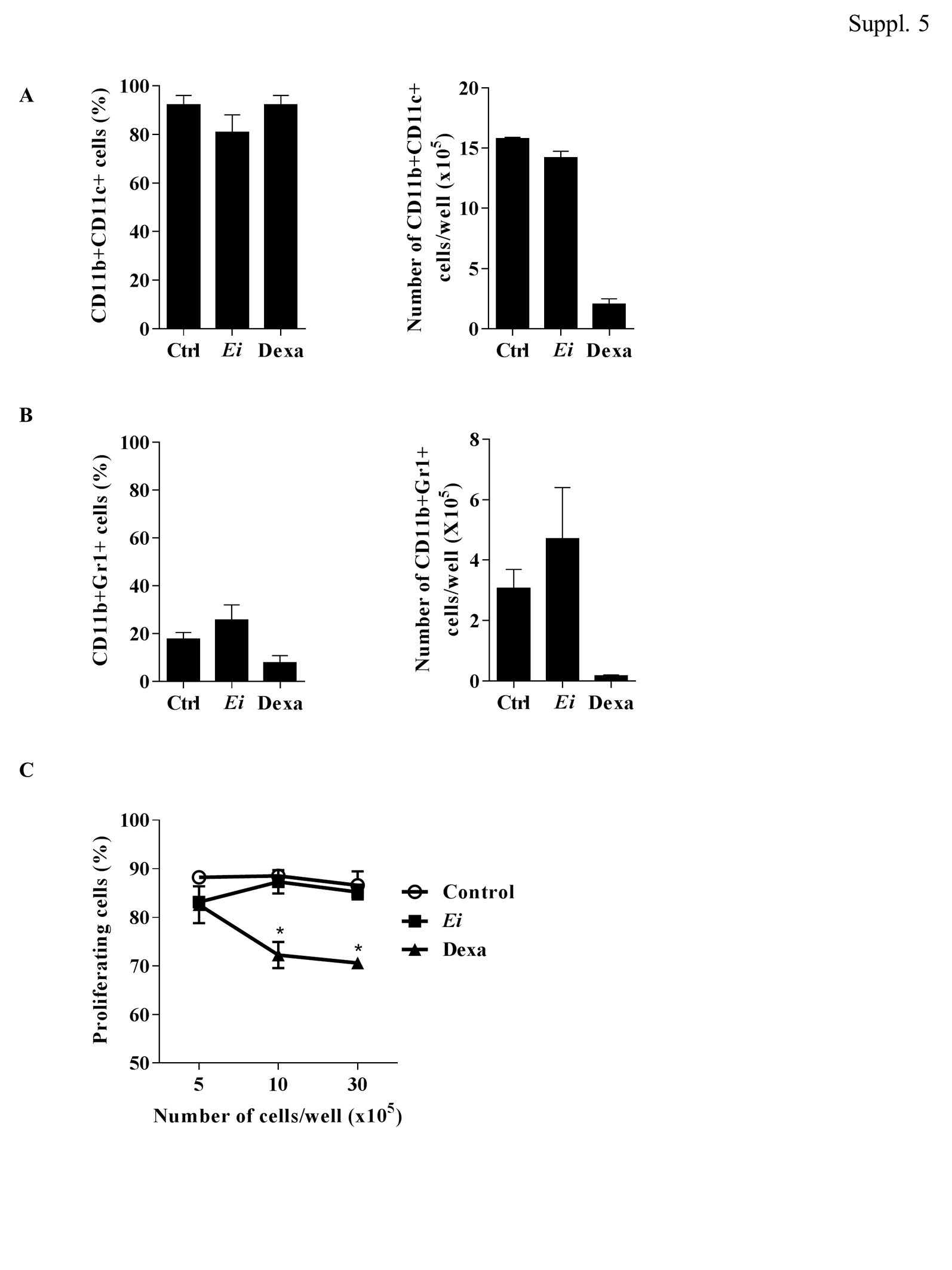

Supplement: Supplementary Figure 5 — Myeloid cell precursors exposed to Ei do not develop into adherent MDSC. BM cells were cultured with GM-CSF for 4 days and Ei spores were added (MOI of 30:1). Non-treated or Dexa-treated cultures (day 4) were set as negative and positive controls, respectively. Cultures were kept in GM-CSF-supplemented culture medium to complete 9 days. Cells in supernatants were then removed and the adherent cells collected, counted and stained with an anti CD11b, CD11c, and Gr1 mAbs to determine de amounts of CD11b+ CD11c+ Gr1- DCs (A) or CD11b+ CD11c- Gr1+ MDSC (B) by flow cytometry. Results are presented as the percentage (left) or the absolute number per well (right). The indicated amounts of cells per well were also co-cultured with polyclonally-activated (anti CD3 mAb/rIL-2) CFSE-labeled naïve lymph node cells during 72 h. The percentage of cells with diluted CFSE was then determined by flow cytometry to assess the suppressive effect (C). Graphs show the mean ±SEM (n = 2 for A and B, n = 4 for C). Student's t-test: *p < 0.05, compared to control. [file Image5.TIF]
